# Centered and Averaged Fuzzy Entropy to Improve Fuzzy Entropy Precision

**DOI:** 10.3390/e20040287

**Published:** 2018-04-15

**Authors:** Jean-Marc Girault, Anne Humeau-Heurtier

**Affiliations:** 1 Groupe ESEO, 49000 Angers, France; 2 Laboratoire Angevin de Recherche en Ingénierie des Systèmes (LARIS), Univ Angers, 49000 Angers, France

**Keywords:** entropy, fuzzy entropy, sample entropy, irregularity, fetal heart rate, time series, symmetrical m-patterns

## Abstract

Several entropy measures are now widely used to analyze real-world time series. Among them, we can cite approximate entropy, sample entropy and fuzzy entropy (FuzzyEn), the latter one being probably the most efficient among the three. However, FuzzyEn precision depends on the number of samples in the data under study. The longer the signal, the better it is. Nevertheless, long signals are often difficult to obtain in real applications. This is why we herein propose a new FuzzyEn that presents better precision than the standard FuzzyEn. This is performed by increasing the number of samples used in the computation of the entropy measure, without changing the length of the time series. Thus, for the comparisons of the patterns, the mean value is no longer a constraint. Moreover, translated patterns are not the only ones considered: reflected, inversed, and glide-reflected patterns are also taken into account. The new measure (so-called centered and averaged FuzzyEn) is applied to synthetic and biomedical signals. The results show that the centered and averaged FuzzyEn leads to more precise results than the standard FuzzyEn: the relative percentile range is reduced compared to the standard sample entropy and fuzzy entropy measures. The centered and averaged FuzzyEn could now be used in other applications to compare its performances to those of other already-existing entropy measures.

## 1. Introduction

Approximate entropy (ApEn) and sample entropy (SampEn) algorithms are now widely used to quantify the irregularity of experimental time series [1,2]. They both rely on the evaluation of vectors’ similarity. However, in both ApEn and SampEn, the vectors’ similarity is based on the Heaviside function, a function that has rigid boundaries. Thus, the contributions of samples inside the boundary are treated equally, but the samples outside the boundary are left out. However, in the real world, boundaries between classes may be ambiguous: it is often difficult to determine if an input pattern belongs totally to a class. To overcome this lack of reality in ApEn and SampEn algorithms, Chen et al. proposed the fuzzy entropy (FuzzyEn) algorithm [3]. In the latter case, the vectors’ similarity is defined by the soft and continuous boundaries of a fuzzy function. Since its introduction, it has been reported that FuzzyEn leads to better performance than ApEn or SampEn [4,5,6]. FuzzyEn presents a stronger relative consistency and shows less dependence on data length than ApEn and SampEn [3].

Nevertheless, the number of samples in a signal still plays a role in the precision of FuzzyEn: the shorter the signal, the lower the number of vectors, and thus, the lower the precision of FuzzyEn (i.e., the larger the standard deviation). Therefore, to obtain more precise entropy values, the longer the signal, the better it is. In practical situations (real data), this may be a challenge. Indeed, it is often difficult to have long recordings, particularly in the biomedical field where patients may have difficulty to stay still or to cooperate.

This is why we herein propose a new fuzzy entropy measure that presents better precision than the traditional FuzzyEn measure. This is performed by increasing the number of samples used in the computation, without changing the length of the time series.

The paper is organized as follows. The original algorithm of FuzzyEn is first detailed in Section 2; then the new entropy measure is described. The synthetic and biomedical data (fetal heart rate time series) used in our work are introduced in Section 3. In Section 4, we first present, analyze, and discuss the results obtained with the synthetic data. We then describe and interpret the results obtained with the biomedical time series. We finally end with the conclusion.

## 2. Standard Fuzzy Entropy and the New Entropy Measure

In this section, we recall the FuzzyEn concept based on the use of a membership function. For this purpose, the generalized Gaussian membership function is used since it allows the derivation of both the rectangular function used in the calculation of SampEn and the standard Gaussian function used in the calculation of FuzzyEn.

### 2.1. Fuzzy Entropy Algorithm

**Figure 1:**
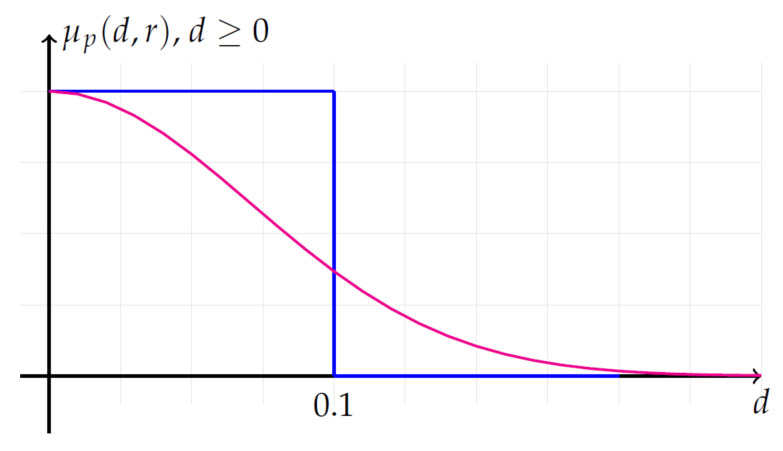
Membership functions $\mu_{p}\left(d,r\right)=\exp\left(-{\left(d/r\right)}^{p}\right)$ with r=0.1. Gaussian function (blue) with p=2; rectangular function (magenta) with p=∞, for d≥0.

For a given discrete time series X={x(1),x(2),…,x(N)} of length *N*, the algorithm to compute FuzzyEn relies on the following steps [1]:

1. Split X into a series of subsequences $X_{m}\left(i\right)$ of length *m* starting at x(i): $X_{m}\left(i\right)=\left\{x\left(i\right),x\left(i+1\right),\ldots ,x\left(i+m-1\right)\right\},1\leq i\leq N-m+1$.
2. For each vector $X_{m}\left(i\right)$, compute the similarity degree $D_{ij}^{m}$ of its neighboring vector $X_{m}\left(j\right)$ using a similarity function as:
  (1) $$D_{ij}^{m}=\mu_{p}\left(d\left[X_{m}\left(i\right),X_{m}\left(j\right)\right],r\right),$$
  where the membership function $\mu_{p}$ reported in Figure 1 is defined ∀d≥0 as:
  (2) $$\mu_{p}\left(d,r\right)=\exp\left(-{\left(d/r\right)}^{p}\right),$$
  and where the distance function *d* is the maximum absolute difference $d\left[X_{m}\left(i\right),X_{m}\left(j\right)\right]=\max_{0\leq k\leq m-1}(|x\left(i+k\right)-x\left(j+k\right)|)$. For p=2, we have the Gaussian function, and for p=∞, we have the rectangular function.
3. For each *i* (1≤i≤N−m+1), compute $\phi_{i}^{m}$ as:
  (3) $$\phi_{i}^{m}\left(r\right)=\frac{1}{N-m-1}\sum_{j=1,j\neq i}^{N-m}D_{ij}^{m}.$$
4. Construct $\varphi^{m}$ and $\varphi^{m+1}$ as:
  (4) $$\varphi^{m}\left(r\right)=\frac{1}{N-m}\sum_{i=1}^{N-m}\phi_{i}^{m}\left(r\right),$$
  (5) $$\varphi^{m+1}\left(r\right)=\frac{1}{N-m}\sum_{i=1}^{N-m}\varphi_{i}^{m+1}\left(r\right).$$
5. Fuzzy entropy is then calculated as:
  (6) $$FuzzyEn\left(m,r\right)=\lim_{N\to \infty }\ln\left[\frac{\varphi^{m}\left(r\right)}{\varphi^{m+1}\left(r\right)}\right],$$
  which, for finite datasets, can be estimated by the statistic:
  (7) $$FuzzyEn\left(m,r,N\right)=\ln\left[\frac{\varphi^{m}\left(r\right)}{\varphi^{m+1}\left(r\right)}\right].$$

**Figure 2:**
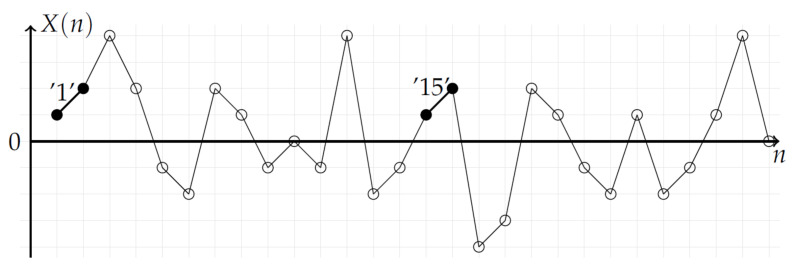
Stochastic time series where 2-patterns are pointed out. Each number corresponds to the place of the corresponding segment. No-centered 2-patterns are considered. The two 2-patterns ‘1’ and ‘15’ (black bullets) have the same mean value; they are similar. The total number of similar 2-patterns is 12: (‘1’,‘15’), (‘5’,‘21’), (‘7’,‘19’), (‘8’,‘20’), (‘13’,‘24’), (‘14’,‘25’).

As shown in Figure 2, the 2-pattern ‘1’ has only one similar 2-pattern among the 27 possible 2-patterns in the time series. From the time series reported in Figure 2, the total number of similar 2-patterns is 12: (‘1’,‘15’), (‘5’,‘21’), (‘7’,‘19’), (‘8’,‘20’), (‘13’,‘24’), (‘14’,‘25’).

As for ApEn and SampEn, the statistical stability of the FuzzyEn estimation depends on the length *N* of the time series as reported in Equation (7). To decrease this length-dependency, several strategies can be proposed.

### 2.2. New Approaches

**Figure 3:**
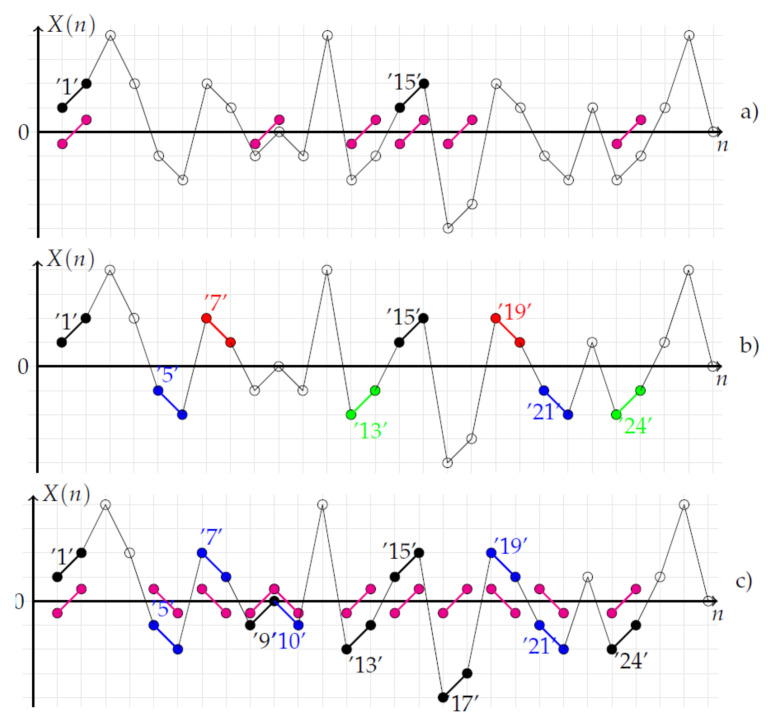
Stochastic time series with different types of 2-patterns. (**a**) Centered 2-patterns are considered. Centered 2-patterns similar to ‘1’ are represented with magenta bullets; there are six patterns similar to ‘1’. The total number of centered similar 2-patterns is 25: (‘1’,‘9’,‘13’,‘15’,‘17’,‘24’), (‘2’,‘14’,‘25’), (‘3’,‘8’,‘20’), (‘4’,‘23’), (‘5’,‘7’,‘10’,‘19’,‘21’), (‘11’,‘18’), (‘12’,‘16’), (‘22’,‘26’). The total number of similar centered 2-patterns is much larger than that of no-centered 2-patterns. (**b**) Regarding the 2-pattern (‘1’), four kinds of 2-patterns can be obtained: 2-patterns with translation (‘T’) in black (‘1’,‘15’), 2-patterns with vertical reflection (‘R’) in red (‘7’, ’19’), 2-patterns with inversion (‘I’) in green (‘13’,‘24’), 2-patterns with glide reflection (‘G’) in blue (‘5’,‘21’). By considering all 2-patterns ranging from ‘1’–‘27’, the mean total number of symmetrical 2-patterns is $N_{sym}=92$ with $N_{sym}^{T}=12$, $N_{sym}^{R}=30$, $N_{sym}^{I}=24$, $N_{sym}^{G}=26$. (**c**) Regarding the 2-pattern (‘1’), two kinds of centered 2-patterns can be obtained: 2-patterns (‘T’,‘I’) in black (‘1’,‘9’,‘13’,‘15’,‘17’,‘24’), 2-patterns (‘R’,‘G’) in blue (‘5’,‘7’,‘10’,‘19’,‘21’). By considering all 2-patterns ranging from ‘1’–‘27’, the mean total number of symmetrical 2-patterns is $Nc_{sym}=312$ with $Nc_{sym}^{T}=86$, $Nc_{sym}^{R}=70$, $Nc_{sym}^{I}=86$ and $Nc_{sym}^{G}=70$.

As mentioned above, from a fixed number of samples *N* in the time series, a way to improve the statistical stability of the entropy measurement consists in artificially increasing the number of similar *m*-patterns taken into account in the entropy calculation. To do so, three different ways are proposed:

1. The first approach is inspired by [3,7]. In the latter studies, the interest in centering each *m*-pattern has been shown. In this case, instead of limiting the search of *m*-patterns with the same mean value, any pattern can be taken into account. Therefore, the number of similar patterns drastically increases.
  Therefore, in the first approach, a centered *m*-pattern $Xc_{m}\left(j\right)$ is compared to a reference centered *m*-pattern $Xc_{m}\left(i\right)$. The similarity degree is calculated with $Xc_{m}\left(i\right)=\left\{x\left(i\right),x\left(i+1\right),\ldots ,x\left(i+m-1\right)\right\}-x_{0}\left(i\right)$, where 1≤i≤N−m+1 and $x_{0}\left(i\right)=\frac{1}{m}\sum_{j=0}^{j=m-1}x\left(i+j\right)$, through a similarity function:
  (8) $$Dc_{ij}^{m}=\mu_{p}\left(d\left[Xc_{m}\left(i\right),Xc_{m}\left(j\right)\right],r\right),$$
  with the same membership function as the one reported in Equation (2). The centered fuzzy entropy FuzzyEn${}_{c}$ is thus defined as:
  (9) $$FuzzyEn_{c}\left(m,r,N\right)=\ln\left[\frac{\varphi_{c}^{m}\left(r\right)}{\varphi_{c}^{m+1}\left(r\right)}\right],$$
  with $\varphi_{c}^{m}\left(r\right)=\frac{1}{N-m}\sum_{i=1}^{N-m}\phi_{ci}^{m}\left(r\right)$ and with $\phi_{ci}^{m}\left(r\right)=\frac{1}{N-m-1}\sum_{j=1,j\neq i}^{N-m}Dc_{ij}^{m}$.
  As shown in Figure 3a, removing the mean value of 2-patterns increases the number of centered similar 2-patterns since the number of centered 2-patterns similar to ‘1’ is six compared to one when the centering approach is not used. From Figure 3b, the total number of centered similar 2-patterns is 25: (‘1’,‘9’,‘13’,‘15’,‘17’,‘24’), (‘2’,‘14’,‘25’), (‘3’,‘8’,‘20’), (‘4’,‘23’), (‘5’,‘7’,‘10’,‘19’,‘21’), (‘11’,‘18’), (‘12’,‘16’), (‘22’,‘26’). The total number of similar centered 2-patterns is much larger than no-centered 2-patterns.
2. The second approach is inspired by [8], where transformed patterns are compared to reference patterns. Thus, in the second approach, a transformed *m*-pattern $\Gamma_{k}\left[X_{m}\left(j\right)\right]$ (see below) is compared to a reference *m*-pattern $X_{m}\left(i\right)$. The similarity degree is calculated with the same membership function as the one reported in Equation (2):
  (10) $${}^{k}D_{ij}^{m}=\mu_{p}\left(d\left[X_{m}\left(i\right),\Gamma_{k}\left[X_{m}\left(j\right)\right]\right],r\right).$$
  Four types of $\Gamma_{k}\left[X_{m}\left(j\right)\right]$ operations with k={T,R,I,G} are evaluated:
  - $\Gamma_{T}\left[X_{m}\left(j\right)\right]=X_{m}\left(j+n\right)$ corresponds to a translation of *n* samples, k=T;
  - $\Gamma_{R}\left[X_{m}\left(j\right)\right]=X_{m}\left(-j+n\right)$ corresponds to a reflection at the position *n*, k=R;
  - $\Gamma_{I}\left[X_{m}\left(j\right)\right]=-X_{m}\left(-j+n\right)$ corresponds to an inversion at the position *n*, k=I;
  - $\Gamma_{G}\left[X_{m}\left(j\right)\right]=-X_{m}\left(j+n\right)$ corresponds to a glide reflection of *n* samples, k=G.
  At first sight, any type of operation could be used. However, from our point of view, only isometries (translation **T**, reflection **R**, inversion **I** and glide reflection **G**) are suitable. This statement is supported by the recent work reported in [8] where the concept of symmetry was placed back on stage in the study of time series. Indeed, in [8], it was shown that the concept of recurrences could be generalized by taking into account the symmetry properties of *m*-patterns. As entropy can be derived from the recurrence concept (the recurrence plot [9] is defined as $RP=\left(N-m+1\right)\sum D_{ij}$ with $\mu_{\infty }\left(d,r\right)$), from [8], four new kinds of entropy ($ApEn_{T}$, $ApEn_{R}$, $ApEn_{I}$, $ApEn_{G}$ or $SampEn_{T}$, $SampEn_{R}$, $SampEn_{I}$, $SampEn_{G}$ or $FuzzyEn_{T}$, $FuzzyEn_{R}$, $FuzzyEn_{I}$, $FuzzyEn_{G}$) can be proposed. Finally, as our ultimate goal is to increase the precision of FuzzyEn, it is more appropriate here to calculate the mean value of the four new fuzzy entropies. In this case, the averaged fuzzy entropy FuzzyEn${}_{a}$ is defined as:
  $$FuzzyEn_{a}\left(m,r,N\right)=\frac{\left(FuzzyEn_{T}+FuzzyEn_{R}+FuzzyEn_{I}+FuzzyEn_{G}\right)}{4},$$
  with:
  $$FuzzyEn_{k}\left(m,r,N\right)=\ln\left[\frac{\varphi_{k}^{m}\left(r\right)}{\varphi_{k}^{m+1}\left(r\right)}\right],$$
  with k={T,R,I,G} for $\varphi_{k}^{m}\left(r\right)=\frac{1}{N-m}\sum_{i=1}^{N-m}\phi_{ki}^{m}\left(r\right)$ and $\phi_{ki}^{m}\left(r\right)=\frac{1}{N-m-1}\sum_{j=1,j\neq i}^{N-m}{}^{k}D_{ij}^{m}$. $FuzzyEn_{T}$ corresponds to the standard FuzzyEn measure when m>1.
  As shown in Figure 3b, the transformation of the 2-patterns increases the number of similar 2-patterns. From Figure 3b, for the 2-pattern (‘1’), four kinds of 2-patterns can be obtained: 2-patterns with translation (‘T’) in black (‘1’,‘15’), 2-patterns with vertical reflection (‘R’) in red (‘7’,‘19’), 2-patterns with inversion (‘I’) in green (‘13’,‘24’) and 2-patterns with glide reflection (‘G’) in blue (‘5’,‘21’). By considering all 2-patterns ranging from ‘1’–‘27’, the mean total number of symmetrical 2-patterns is $N_{sym}=92$ with $N_{sym}^{T}=12$, $N_{sym}^{R}=30$, $N_{sym}^{I}=24$, $N_{sym}^{G}=26$.
3. The last approach compares a centered *m*-pattern $Xc_{m}\left(i\right)$ to a transformed centered *m*-pattern $\Gamma_{k}\left[Xc_{m}\left(j\right)\right]$. In this case, the centered and averaged fuzzy entropy FuzzyEn${}_{\mathrm{ca}}$ is defined as:
  $$FuzzyEn_{ca}\left(m,r,N\right)=\frac{\left(FuzzyEn_{cT}+FuzzyEn_{cR}+FuzzyEn_{cI}+FuzzyEn_{cG}\right)}{4},$$
  with:
  $$FuzzyEn_{ck}\left(m,r,N\right)=\ln\left[\frac{\varphi_{ck}^{m}\left(r\right)}{\varphi_{ck}^{m+1}\left(r\right)}\right],$$
  with k={T,R,I,G} for $\varphi_{ck}^{m}\left(r\right)=\frac{1}{N-m}\sum_{i=1}^{N-m}\phi_{cki}^{m}\left(r\right)$ and $\phi_{cki}^{m}\left(r\right)=\frac{1}{N-m-1}\sum_{j=1,j\neq i}^{N-m}{}^{k}Dc_{ij}^{m}$. ${}^{k}Dc_{ij}^{m}$ is defined as ${}^{k}Dc_{ij}^{m}=\mu_{p}\left(d\left[Xc_{m}\left(i\right),\Gamma_{k}\left[Xc_{m}\left(j\right)\right]\right],r\right)$.
  As shown in Figure 3, one can observe that the combination of the centering and averaging operations globally increases the number of *m*-patterns taken into account in the calculation of the entropy measure. Furthermore, a centered *m*-pattern transformed by an inversion (‘I’) is similar to a centered *m*-pattern transformed by a translation (’T’). The same remark applies for glide and vertical reflection transformations of centered *m*-patterns.
  From Figure 3c, regarding the 2-pattern (‘1’), two kinds of centered 2-patterns can be obtained: 2-patterns (‘T’,‘I’) in black (‘1’,‘9’,‘13’,‘15’,‘17’,‘24’) and 2-patterns (‘R’,‘G’) in blue (‘5’,‘7’,‘10’,‘19’,‘21’). By considering all 2-patterns ranging from ‘1’–‘27’, the mean total number of symmetrical 2-patterns is $Nc_{sym}=312$ with $Nc_{sym}^{T}=86$, $Nc_{sym}^{R}=70$, $Nc_{sym}^{I}=86$ and $Nc_{sym}^{G}=70$.

The novelty of our method therefore relies on two main points: (i) the mean value of the patterns is no longer a constraint in the computation as the patterns are centered; (ii) translated patterns, but also reflected, inversed, and glide-reflected patterns are taken into account (in the standard sample and fuzzy entropy measures, only translated patterns are considered). Therefore, for a given number of samples *N* in the time series, we managed to increase the number of similar *m*-patterns taken into account in the entropy calculation. In what follows, the new entropy measure will be applied to synthetic $1/f^{\beta }$ time series and biomedical datasets. Its precision will be compared to the one of the standard FuzzyEn.

## 3. Data Processed

### 3.1. Synthetic Signals

In order to analyze the new fuzzy entropy measures and to compare their performances with the ones of the standard FuzzyEn, we used $1/f^{\beta }$ time series, with different β values: β varied from −1 to 2 in steps of 0.2. For β>0, the $1/f^{\beta }$ signals are persistent processes with long-term correlations [10]. However, for β<0, the $1/f^{\beta }$ signals are anti-persistent processes with short-term anti-correlations [10]. From a theoretical point of view, the higher the value of β, the larger the number of correlations in the time series and, therefore, the larger the number of similar samples used in the computation of FuzzyEn. For each β value, 50 time series were simulated.

### 3.2. Biomedical Data

The new descriptors mentioned above were also applied to biomedical data and more precisely to fetal heart rate (FHR) time series. The latter were acquired using a homemade pulse Doppler system co-developed with Altaïs Technologies (Tours, France). This Doppler fetal monitor transmits ultrasound waves of 2.25 MHz for an acoustic power limited to 1 mW/cm${}^{2}$ (for more details, see [11]). It was developed to measure both the FHR and fetal movements (pseudo-breathing, limb movements).

The study was approved by the Ethics Committee of the Clinical Investigation Centre for Innovative Technology of Tours (CIC-IT 806 CHRUof Tours). Before acquisition, the consent of each parent was obtained. All parents were over eighteen years of age, and pregnancies were single. After locating the fetal heart with an echographic scanner, 18 Doppler recordings of 30 min each were acquired at CHRU Bretonneau Tours, France. This corresponds to approximately 3600 heart beats for each recording. In order to constitute homogeneous groups without spurious data, gestations complicated by other kinds of disorders (hypertension, diabetes) were discarded. Two groups of fetuses were selected: normal and those with severe intra-uterine growth retardation (IUGR). The severe IUGR group included nine fetuses delivered prematurely by cesarean section. The normal group included nine fetuses without disorders, delivered at term by spontaneous labor. For this clinical protocol, the gestational ages of fetuses ranged from 30–34 weeks. 

In what follows, the 30 min of data were processed, but also segments of 10 min and 20 min. Our goal was thus to compare the results obtained as the data length decreases. Moreover, in order to compare the results obtained between normal and IUGR groups, a Mann–Whitney test was used. A *p*-value strictly less than 0.05 was considered to define statistical significance.

## 4. Results and Discussion

In all that follows, the value of *r* is set at 0.1 × the standard deviation of the time series.

### 4.1. Results for the Synthetic Signals

In order to validate our hypothesis (that is, the greater the number of similar *m*-patterns taken into account in the computation, the more precise the entropy measure), we started by counting the number of similar *m*-patterns from 50 synthetic time series.

From $1/f^{\beta }$ noises generated with N=5000 samples with β ranging from −1 to 2, the median of the mean number MN of similar 3-patterns and the median of the mean number $MN_{ca}$ of centered and averaged similar 3-patterns were evaluated and are reported in Table 1. As expected, the higher the sample correlation in the time series, the higher the value of β and the higher the number of similar 3-patterns. Indeed, from Table 1, when β increases from 0 to 2, MN goes from 1 to 162. When symmetrical properties and the centering operation are taken into account, $MN_{ca}$ goes from 21 to 9278 for β ranging from 0 to 2. From this, it can be claimed that the averaging and the centering operations increase the number of similar patterns. Furthermore, whatever the *m*-value, we obtain rising trends as β increases (data not shown).

In order to evaluate the performance of our new approaches, for a fixed *m*-value and for 50 $1/f^{\beta }$ time series with different β values, different measures have been computed: the medians MFuzzyEn, $MFuzzyEn_{c}$, $MFuzzyEn_{a}$, $MFuzzyEn_{ca}$ and the percentiles at 75% and 25% PFuzzyEn(75), PFuzzyEn(25), $PFuzzyEn_{c}\left(75\right)$, $PFuzzyEn_{c}\left(25\right)$, $PFuzzyEn_{a}\left(75\right)$, $PFuzzyEn_{a}\left(25\right)$, $PFuzzyEn_{ca}\left(75\right)$, $PFuzzyEn_{ca}\left(25\right)$ have been compared.

To quantitatively evaluate the gain brought by our new approaches in comparison with FuzzyEn, two kinds of statistics have been evaluated: percentile ranges and relative percentile ranges. The following percentile ranges have thus been computed:

- $R_{F}=PFuzzyEn\left(75\right)-PFuzzyEn\left(25\right)$;
- $R_{Fc}=PFuzzyEn_{c}\left(75\right)-PFuzzyEn_{c}\left(25\right)$;
- $R_{Fa}=PFuzzyEn_{a}\left(75\right)-PFuzzyEn_{a}\left(25\right)$;
- $R_{Fca}=PFuzzyEn_{ca}\left(75\right)-PFuzzyEn_{ca}\left(25\right)$.

Finally, from the percentile ranges, the following relative percentile ranges have been evaluated:

- $\left(R_{F}-R_{Fc}\right)/R_{Fc}$;
- $\left(R_{F}-R_{Fa}\right)/R_{Fa}$;
- $\left(R_{F}-R_{Fca}\right)/R_{Fca}$.

**Figure 4:**
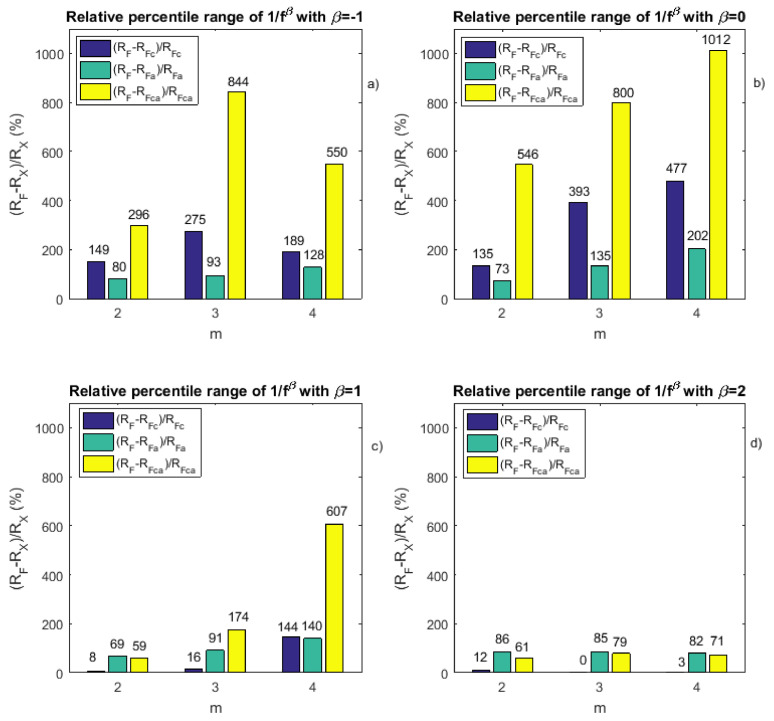
Relative percentile ranges derived from Table A1, Table A2 and Table A3 reported in the Appendix. (**a**) For β=−1, relative percentile range values obtained for different *m*-values: for the centered fuzzy entropy compared to the fuzzy entropy ($\left(R_{F}-R_{Fc}\right)/R_{Fc}$), for the averaged fuzzy entropy compared to the fuzzy entropy ($\left(R_{F}-R_{Fa}\right)/R_{Fa}$) and for the centered and averaged fuzzy entropy compared to the fuzzy entropy ($\left(R_{F}-R_{Fca}\right)/R_{Fca}$); (**b**–**d**) similar to (**a**), but for β=0, β=1 and β=2, respectively.

The global results are presented in Table A1, Table A2 and Table A3 reported in the Appendix and are shown in Figure 4. We observe from the tables that SampEn leads to worse results than FuzzyEn, as already shown by others. Moreover, we observe that the new approach leads to results that show a reduced percentile range compared to the standard fuzzy entropy measure. Its precision is therefore better than the other entropy measures. However, our work also has some drawbacks: the gain provided by the method depends on the signal properties. The gain differs with β values.

### 4.2. Results for the Fetal Heart Rate Time Series

**Figure 5:**
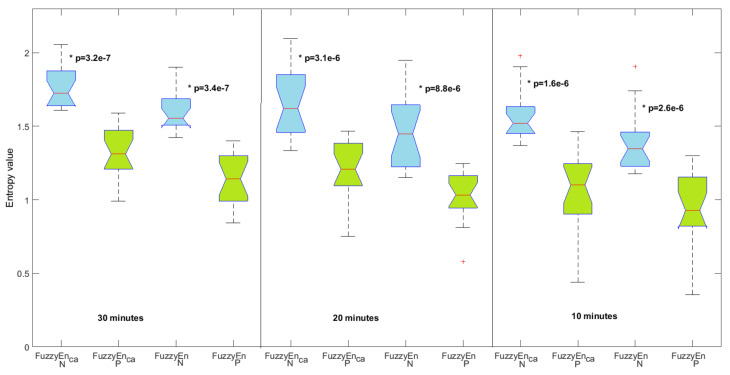
Centered and averaged fuzzy entropy ($FuzzyEn_{ca}$) and standard fuzzy entropy (FuzzyEn) for normal (N) in blue and pathological fetuses (P) in green with m=2. The results for three data lengths are shown. ${}^{⋆}$ means statistically significant between the two groups.

The results obtained from FHR time series for m=2 are presented in Figure 5 for data lengths of 10 min, 20 min, and 30 min. For the three data lengths, we observe that the normal fetuses show a significantly higher entropy value than the pathological fetuses. This is true for the two entropy measures: $FuzzyEn_{ca}$ and the standard FuzzyEn. This means that FHR time series are more irregular for the normal fetuses than for the pathological ones. We also observe that the *p*-value between the two groups decreases as the data length increases. Therefore, the longer the data, the better the separation between the two groups. However, we note that, whatever the length studied, the *p*-value is lower for $FuzzyEn_{ca}$ than for the standard FuzzyEn. Our new entropy measure is therefore more interesting for this classification purpose than the standard FuzzyEn. Other data may now be processed; see, e.g., [12,13,14].

## 5. Conclusions

A new entropy measure, $FuzzyEn_{\mathrm{ca}}$, is proposed to improve the precision of the standard FuzzyEn. The new measure relies on centering and averaging approaches that lead to a larger number of similar patterns used in the computation of the entropy algorithm. This is performed by removing the constraint of the mean value in the comparison of the patterns. Moreover, translated patterns are not the only ones considered: reflected, inversed, and glide-reflected patterns are also taken into account. The results obtained on $1/f^{\beta }$ time series reveal that $FuzzyEn_{\mathrm{ca}}$ shows a greater precision than FuzzyEn. Moreover, when applied to FHR time series acquired from normal and pathological fetuses, $FuzzyEn_{\mathrm{ca}}$ leads to a better discrimination between the two groups than the standard FuzzyEn. These findings could allow one to obtain entropy-based relevant information by processing shorter datasets (we could obtain the same precision as the standard FuzzyEn, but with less data). This is particularly interesting for the biomedical field. $FuzzyEn_{\mathrm{ca}}$ now has to be applied to other datasets, and its performance has to be compared to those of other already-existing entropy measures.

## Figures and Tables

**Table 1:** For the calculation of FuzzyEn and $FuzzyEn_{ca}$, the median of the mean number MN of similar 3-patterns and the median of the mean number of centered and averaged $MN_{ca}$ of similar 3-patterns obtained from $1/f^{\beta }$ noises (N=5000 samples) with β ranging from −1 to 2. $MN_{ca}=\left(MN_{ca}^{T}+MN_{ca}^{R}+MN_{ca}^{I}+MN_{ca}^{G}\right)$, where $MN_{ca}^{k}$ is the median of the number of centered symmetric similar 3-patterns obtained in the calculation of $FuzzyEn_{ca}$, $k={\{}^{\prime }T^{\prime },\prime R^{\prime },\prime I^{\prime },\prime G^{\prime }\}$. For the computation, m=3 and r=0.1× standard deviation of the time series.

β	−1	−0.8	−0.6	−0.4	−0.2	0	0.2	0.4	0.6	0.8	1.0	1.2	1.4	1.6	1.8	2.0
MN	0.73	0.69	0.65	0.62	0.6	0.63	0.63	0.67	0.80	1.07	1.76	3.64	9.24	26.47	71.68	162.38
$MN_{ca}$	16.71	17.03	17.48	18.13	19.18	20.75	23.19	27.35	35.71	53.15	93.73	206.09	540.00	1580.67	4317.40	9277.86

## Appendix A

Results reported in Table A1, Table A2 and Table A3 show performances that differ with the β values. We observe that the higher the β value, the lower the gain obtained in terms of relative percentile range. This is probably due to the level of correlation between samples in the time series.

**Table A1:** Results obtained for $1/f^{\beta }$ time series, for m=2,N=5000 samples and for different β values.

β value	−1	−0.8	−0.6	−0.4	−0.2	0	0.2	0.4	0.6	0.8	1	1.2	1.4	1.6	1.8	2
MSampEn	3.46	3.5	3.53	3.54	3.57	3.59	3.58	3.54	3.45	3.29	3.04	2.68	2.21	1.68	1.13	0.64
MFuzzyEn	3.17	3.21	3.25	3.26	3.28	3.28	3.28	3.25	3.16	3.00	2.76	2.40	1.93	1.42	0.93	0.54
$MFuzzyEn_{c}$	3.58	3.58	3.58	3.58	3.56	3.53	3.49	3.42	3.30	3.11	2.84	2.46	1.99	1.46	0.94	0.51
$MFuzzyEn_{a}$	3.14	3.17	3.2	3.22	3.24	3.24	3.23	3.20	3.12	2.97	2.73	2.37	1.90	1.40	0.93	0.57
$MFuzzyEn_{ca}$	3.57	3.58	3.58	3.57	3.56	3.53	3.48	3.41	3.30	3.11	2.83	2.44	1.96	1.44	0.94	0.53
RSampEn	0.05	0.08	0.07	0.08	0.09	0.08	0.08	0.10	0.08	0.05	0.08	0.11	0.16	0.24	0.25	0.23
RFuzzyEn	0.03	0.04	0.03	0.04	0.04	0.04	0.04	0.05	0.04	0.04	0.08	0.11	0.17	0.22	0.22	0.18
$RFuzzyEn_{c}$	0.01	0.01	0.01	0.01	0.01	0.02	0.02	0.01	0.02	0.03	0.07	0.11	0.18	0.23	0.23	0.20
$RFuzzyEn_{a}$	0.02	0.03	0.02	0.02	0.02	0.02	0.02	0.03	0.03	0.02	0.04	0.08	0.11	0.13	0.13	0.10
$RFuzzyEn_{ca}$	0.01	0.01	0.01	0.01	0.01	0.01	0.01	0.01	0.01	0.02	0.05	0.08	0.11	0.14	0.14	0.11
(RSampEn−RFuzzyEn)/RFuzzyEn	0.63	0.75	1.24	1.09	1.17	1.04	1.10	0.99	0.90	0.43	0.12	0.00	-0.05	0.10	0.16	0.29
$\left(RSampEn-RFuzzyEn_{c}\right)/RFuzzyEn_{c}$	3.08	5.77	5.6	7.06	6.11	3.80	4.27	5.85	3.17	0.68	0.21	-0.05	-0.12	0.08	0.09	0.14
$\left(RSampEn-RFuzzyEn_{a}\right)/RFuzzyEn_{a}$	1.95	2.09	3.26	3.34	4.38	2.53	2.78	2.72	2.03	1.33	0.89	0.40	0.50	0.89	1.03	1.39
$\left(RSampEn-RFuzzyEn_{ca}\right)/RFuzzyEn_{ca}$	5.47	8.67	9.1	9.69	11.3	12.20	8.96	8.57	5.55	1.59	0.77	0.36	0.43	0.74	0.86	1.07
$\left(RFuzzyEn-RFuzzyEn_{c}\right)/RFuzzyEn_{c}$	1.49	2.86	1.95	2.87	2.27	1.35	1.51	2.44	1.19	0.18	0.08	0.05	0.07	0.02	0.07	0.12
$\left(RFuzzyEn-RFuzzyEn_{a}\right)/RFuzzyEn_{a}$	0.8	0.76	0.91	1.08	1.48	0.73	0.80	0.87	0.59	0.63	0.69	0.40	0.58	0.72	0.75	0.86
$\left(RFuzzyEn-RFuzzyEn_{ca}\right)/RFuzzyEn_{ca}$	2.96	4.51	3.52	4.13	4.66	5.46	3.74	3.81	2.44	0.81	0.59	0.36	0.51	0.59	0.60	0.61

**Table A2:** Same as Table A1, but for m=3.

β value	−1	−0.8	−0.6	−0.4	−0.2	0	0.2	0.4	0.6	0.8	1	1.2	1.4	1.6	1.8	2
MSampEn	3.47	3.51	3.48	3.54	3.56	3.57	3.54	3.58	3.43	3.27	3.01	2.65	2.18	1.66	1.13	0.64
MFuzzyEn	3.02	3.1	3.12	3.15	3.2	3.18	3.16	3.15	3.04	2.88	2.61	2.26	1.80	1.30	0.84	0.49
$MFuzzyEn_{c}$	3.28	3.27	3.28	3.29	3.27	3.26	3.22	3.16	3.05	2.87	2.59	2.22	1.77	1.26	0.80	0.44
$MFuzzyEn_{a}$	2.99	3.02	3.04	3.07	3.11	3.10	3.10	3.05	2.97	2.82	2.58	2.22	1.77	1.29	0.84	0.51
$MFuzzyEn_{ca}$	3.21	3.22	3.23	3.24	3.23	3.22	3.18	3.12	3.02	2.85	2.59	2.21	1.74	1.25	0.80	0.47
RSampEn	0.51	0.49	0.43	0.5	0.66	0.58	0.59	0.39	0.40	0.35	0.19	0.18	0.18	0.21	0.25	0.23
RFuzzyEn	0.17	0.16	0.13	0.17	0.23	0.17	0.18	0.14	0.17	0.11	0.09	0.11	0.17	0.19	0.20	0.16
$RFuzzyEn_{c}$	0.05	0.04	0.03	0.03	0.05	0.03	0.04	0.04	0.03	0.05	0.08	0.11	0.17	0.20	0.20	0.16
$RFuzzyEn_{a}$	0.09	0.07	0.08	0.07	0.06	0.07	0.08	0.07	0.06	0.06	0.05	0.07	0.10	0.13	0.12	0.09
$RFuzzyEn_{ca}$	0.02	0.02	0.02	0.02	0.02	0.02	0.02	0.02	0.02	0.02	0.03	0.08	0.11	0.13	0.12	0.09
(RSampEn−RFuzzyEn)/RFuzzyEn	1.95	2.13	2.46	1.86	1.94	2.48	2.23	1.78	1.38	2.21	1.15	0.54	0.08	0.08	0.25	0.42
$\left(RSampEn-RFuzzyEn_{c}\right)/RFuzzyEn_{c}$	10.08	11.6	11.8	14.54	12.6	16.19	14.51	8.33	11.03	5.97	1.49	0.58	0.05	0.03	0.25	0.41
$\left(RSampEn-RFuzzyEn_{a}\right)/RFuzzyEn_{a}$	4.68	5.55	4.51	5.74	9.74	7.20	6.06	4.93	6.25	4.90	3.12	1.60	0.74	0.64	1.17	1.62
$\left(RSampEn-RFuzzyEn_{ca}\right)/RFuzzyEn_{ca}$	26.88	22.74	17.41	28.43	33.81	30.33	30.39	21.08	23.78	16.41	4.91	1.34	0.59	0.57	1.09	1.55
$\left(RFuzzyEn-RFuzzyEn_{c}\right)/RFuzzyEn_{c}$	2.75	3.03	2.7	4.43	3.63	3.93	3.80	2.36	4.05	1.17	0.16	0.03	0.03	0.05	0.00	0.00
$\left(RFuzzyEn-RFuzzyEn_{a}\right)/RFuzzyEn_{a}$	0.92	1.1	0.59	1.36	2.65	1.35	1.18	1.13	2.05	0.84	0.91	0.69	0.61	0.52	0.74	0.85
$\left(RFuzzyEn-RFuzzyEn_{ca}\right)/RFuzzyEn_{ca}$	8.44	6.59	4.32	9.28	10.84	8.00	8.71	6.94	9.41	4.42	1.74	0.52	0.47	0.45	0.68	0.79

**Table A3:** Same as Table A1, but for m=4. “-” means that an undefined value is obtained due the absence of similar *m*-patterns in the time series.

β value	−1	−0.8	−0.6	−0.4	−0.2	0	0.2	0.4	0.6	0.8	1	1.2	1.4	1.6	1.8	2
MSampEn	-	-	-	-	-	-	-	-	-	-	2.95	2.71	2.19	1.59	1.13	0.64
MFuzzyEn	3.09	3.02	2.95	3.08	3.01	3.26	3.17	3.13	2.99	2.75	2.50	2.13	1.70	1.17	0.78	0.45
$MFuzzyEn_{c}$	3.15	3.15	3.14	3.15	3.19	3.14	3.13	3.08	2.95	2.79	2.51	2.11	1.70	1.16	0.76	0.43
$MFuzzyEn_{a}$	2.73	2.73	2.84	2.77	2.81	2.92	2.84	2.87	2.72	2.55	2.35	2.05	1.65	1.19	0.78	0.47
$MFuzzyEn_{ca}$	3.08	3.11	3.12	3.11	3.11	3.12	3.09	3.04	2.93	2.76	2.51	2.14	1.67	1.19	0.76	0.44
RSampEn	-	-	-	-	-	-	-	-	-	-	1.09	0.49	0.26	0.28	0.26	0.22
RFuzzyEn	0.61	0.39	0.71	0.59	0.58	0.80	0.63	0.58	0.29	0.40	0.22	0.18	0.16	0.21	0.19	0.15
$RFuzzyEn_{c}$	0.21	0.16	0.16	0.15	0.12	0.14	0.12	0.11	0.08	0.06	0.09	0.14	0.16	0.23	0.19	0.15
$RFuzzyEn_{a}$	0.27	0.19	0.29	0.28	0.29	0.27	0.29	0.26	0.24	0.19	0.09	0.10	0.10	0.10	0.11	0.08
$RFuzzyEn_{ca}$	0.09	0.1	0.1	0.08	0.07	0.07	0.05	0.06	0.04	0.03	0.03	0.06	0.11	0.10	0.12	0.09
(RSampEn−RFuzzyEn)/RFuzzyEn	-	-	-	-	-	-	-	-	-	-	4.03	1.75	0.64	0.31	0.38	0.49
$\left(RSampEn-RFuzzyEn_{c}\right)/RFuzzyEn_{c}$	-	-	-	-	-	-	-	-	-	-	11.29	2.60	0.59	0.22	0.40	0.44
$\left(RSampEn-RFuzzyEn_{a}\right)/RFuzzyEn_{a}$	-	-	-	-	-	-	-	-	-	-	11.06	4.13	1.54	1.93	1.44	1.70
$\left(RSampEn-RFuzzyEn_{ca}\right)/RFuzzyEn_{ca}$	-	-	-	-	-	-	-	-	-	-	34.58	6.91	1.33	1.92	1.27	1.54
$\left(RFuzzyEn-RFuzzyEn_{c}\right)/RFuzzyEn_{c}$	1.89	1.37	3.38	2.97	3.94	4.77	4.26	4.10	2.76	6.28	1.44	0.31	0.03	0.07	0.01	0.03
$\left(RFuzzyEn-RFuzzyEn_{a}\right)/RFuzzyEn_{a}$	1.28	1.03	1.49	1.13	1	2.02	1.16	1.26	0.23	1.07	1.40	0.86	0.55	1.24	0.77	0.82
$\left(RFuzzyEn-RFuzzyEn_{ca}\right)/RFuzzyEn_{ca}$	5.5	2.79	6.16	6.22	6.89	10.12	11.25	8.05	6.25	12.67	6.07	1.87	0.42	1.23	0.64	0.71
